# Non-invasive Brain Stimulation for Neuropathic Pain After Spinal Cord Injury: A Systematic Review and Network Meta-Analysis

**DOI:** 10.3389/fnins.2021.800560

**Published:** 2022-02-11

**Authors:** Lingling Li, Hailiang Huang, Ying Yu, Yuqi Jia, Zhiyao Liu, Xin Shi, Fangqi Wang, Tingting Zhang

**Affiliations:** ^1^College of Rehabilitation Medicine, Shandong University of Traditional Chinese Medicine, Jinan, China; ^2^Innovative Institute of Chinese Medicine and Pharmacy, Shandong University of Traditional Chinese Medicine, Jinan, China; ^3^College of Chemical Engineering and Technology, Hebei University of Technology, Tianjin, China

**Keywords:** non-invasive brain stimulation, spinal cord injury, neuropathic pain, network meta-analysis, systematic review

## Abstract

**Objective:**

This study aims to systematically evaluate the effect of non-invasive brain stimulation (NIBS) on neuropathic pain (NP) after spinal cord injury and compare the effects of two different NIBS.

**Methods:**

Randomized controlled trials (RCTs) about the effect of NIBS on NP after spinal cord injury (SCI) were retrieved from the databases of PubMed, Embase, Cochrane Library, Web of Science, CNKI, Wanfang Data, VIP, and CBM from inception to September 2021. The quality of the trials was assessed, and the data were extracted according to the Cochrane handbook of systematic review. Statistical analysis was conducted with Stata (version 16) and R software (version 4.0.2).

**Results:**

A total of 17 studies involving 507 patients were included. The meta-analysis showed that NIBS could reduce the pain score (SMD = −0.84, 95% CI −1.27 −0.40, *P* = 0.00) and the pain score during follow-up (SMD = −0.32, 95%CI −0.57 −0.07, *P* = 0.02), and the depression score of the NIBS group was not statistically significant than that of the control group (SMD = −0.43, 95%CI −0.89–0.02, *P* = 0.06). The network meta-analysis showed that the best probabilistic ranking of the effects of two different NIBS on the pain score was repetitive transcranial magnetic stimulation (rTMS) (*P* = 0.62) > transcranial direct current stimulation (tDCS) (*P* = 0.38).

**Conclusion:**

NIBS can relieve NP after SCI. The effect of rTMS on NP is superior to that of tDCS. We suggest that the rTMS parameters are 80–120% resting motion threshold and 5–20 Hz, while the tDCS parameters are 2 mA and 20 min. However, it is necessary to carry out more large-scale, multicenter, double-blind, high-quality RCT to explore the efficacy and mechanism of NIBS for NP after SCI.

## Introduction

The International Association for the Study of Pain (IASP) defines neuropathic pain (NP) as “pain caused by injury or disease of the physical sensory system,” which is mainly characterized by spontaneous pain, hyperalgesia, and abnormal sensation (Colloca et al., 2017). It is one of the most common and challenging complications after spinal cord injury, with a prevalence of 53% (Burke et al., 2017). Some patients have secondary symptoms such as depression and sleep disorders due to pain, which negatively impact the quality of life of patients. Although IASP has recommended NP to be treated with drugs (such as antidepressants, anticonvulsants, local anesthetics, opioids, etc.) (Szczudlik et al., 2014), unfortunately, only 30–50% of patients respond to drug treatment, 60–70% of patients do not receive pain relief (Finnerup et al., 2005; Hansson et al., 2009), and some patients stop the treatment because of the side effects of the drugs. Besides these, invasive electrical stimulation of the motor cortex has been reported to have a certain analgesic effect on NP, but this brain stimulation is expensive and invasive and may produce additional side effects (infection, intracranial hemorrhage, etc.), limiting its clinical application (Defrin et al., 2007).

Non-invasive brain stimulation (NIBS) mainly regulates the excitability of the cerebral cortex through electric fields or magnetic fields, which has the advantages of non-invasive and easy operation and has a broad clinical application prospect (Godinho et al., 2017). Repetitive transcranial magnetic stimulation (rTMS) and transcranial direct current stimulation (tDCS) are two typical methods of NIBS, each of which has its advantages (Bandeira et al., 2021). In the former, the time-varying magnetic field acts on the cerebral cortex to produce induced current, which changes the action potential of cortical neurons, thus affecting brain metabolism and neuroelectric activity (Fisicaro et al., 2019). The latter uses a weak direct current to regulate the activity of cortical neurons in the cerebral cortex. When the anode approaches the nerve in the cell body or dendrite, the neuron discharge increases, while when the direction of the electric field is reversed, the neuron discharge decreases. Anode stimulation increases excitability, while cathode stimulation decreases excitability (Klomjai et al., 2015).

Previous studies have shown that NIBS can relieve NP after spinal cord injury (SCI) (Ngernyam et al., 2015; Sun et al., 2019) compared with the control group, but some studies have shown that NIBS has no obvious therapeutic effect (Defrin et al., 2007; Wrigley et al., 2013). Although some studies have explored the rehabilitation of NIBS for NP after SCI, the sample size of a single research is small, and the inclusion criteria and research methods are different. Because of the lack of evidence-based research on the rehabilitation of NIBS for NP after SCI and the comparison of the effects of two different NIBS, it is not conducive to developing evidence-based clinical practice of NIBS in treating NP after SCI. Therefore, this paper will systematically evaluate the rehabilitation of NIBS on NP after SCI through evidence-based medicine and compare the differences of the effects between two different NIBS to provide some reference and basis for the future application of NIBS in clinical rehabilitation.

## Methods

### Search Strategy

Randomized controlled trials (RCTs) about the effect of NIBS on NP after SCI were retrieved by two researchers (TZ and FW) from PubMed, Embase, Cochrane Library, Web of Science, CNKI, Wanfang Data, VIP, and CBM from inception to September 2021. We searched the databases by Mesh words combined with free words and supplemented the studies by reading relevant reviews and meta-analyses.

Taking the EMBASE database as an example, the specific retrieval strategy is as follows: (“transcranial direct current stimulation”: ti, ab, kw OR “transcranial magnetic stimulation”: ti, ab, kw OR “repetitive transcranial magnetic stimulation”: ti, ab, kw OR “non-invasive brain stimulation”: ti, ab, kw OR “non-invasive brain stimulation”: ti, ab, kw OR “transcranial electrical stimulation”: ti, ab, kw OR rTMS: ti, ab, kw OR tDCS: ti, ab, kw OR NIBS: ti, ab, kw) AND (“spinal cord trauma”: ti, ab, kw OR “SCI”: ti, ab, kw OR “spinal cord transection”: ti, ab, kw OR “spinal cord contusion”: ti, ab, kw OR SCI: ti, ab, kw) AND (neuralgia: ti, ab, kw OR “neuropathic pain”: ti, ab, kw OR “chronic pain”: ti, ab, kw OR “central pain”: ti, ab, kw OR pain: ti, ab, kw).

### Inclusion Criteria

The study participants included patients with NP after SCI. The intervention included NIBS, including tDCS and rTMS. Comparison was carried out on sham–NIBS. The outcome is twofold: (1) primary outcomes, which include Visual Analog Score (VAS), Numerical Rating Scale (NRS), and secondary outcomes, including Beck Depression Inventory (BDI), Hamilton Depression Scale (HAMD), brain-derived neurotrophic factor (BDNF), and nerve growth factor (NGF). The study design was RCTs.

### Exclusion Criteria

Studies that meet the following criteria should be excluded: non-RCTs, protocols, repeated publication, conference abstracts and reviews, without corresponding outcomes, animal experiments, case reports, those with incomplete data, original data, or full-text documents that cannot be obtained after contacting the author, etc.

### Data Extraction

According to the inclusion and exclusion criteria, two researchers (YY and YJ) independently screened the studies, extracted the data, and cross-checked the screened results. If there were differences, they would discuss and solve them or consult a third researcher (Huang). In the screening of studies, reading the title and abstract of the studies was the first course of action. After excluding the irrelevant studies, the full text was read to determine the final study. The extracted contents include the basic information of included studies (first author and publication year), baseline situation (sample size, age, duration, SCI degree, level, etc.), intervention measures (type, intensity, time, frequency, etc.), outcomes, adverse reactions, follow-up data, and quality evaluation information.

### Quality and Risk-of-Bias Assessment

The Cochrane risk-of-bias tool and Physiotherapy Evidence Database were used to assess the methodological quality of the included studies (Sterne et al., 2019; Albanese et al., 2020).

### Statistical Analysis

Meta-analysis was conducted with Stata (version 16). Relative risk ratio was used as the effect size for the two classification variables. Weighted mean difference was used as the effect size for continuous variables, and standard mean difference (SMD) was used when the measurement method or unit was inconsistent. The 95% confidence interval (CI) was calculated, and the test level α was 0.05. Considering the heterogeneity among the included studies, the random effect model was adopted. If there was considerable heterogeneity among the included studies, subgroup analysis, meta-regression, and Hartung–Knapp–Sidik–Jonkman (HKSJ) (IntHout et al., 2014) method should be performed to explore the source of heterogeneity.

R software (version 4.0.2) was used to conduct a network meta-analysis based on Markov Chain Monte Carlo fitting consistency model to compare the efficacy differences between tDCS and rTMS. The convergence was evaluated by the bandwidth value. The bandwidth value was closer to 0, indicating that the convergence was better, and the analysis results of the consistency model were more reliable (Yi et al., 2015).

## Result

### Study Selection and Characteristics

**Figure 1:**
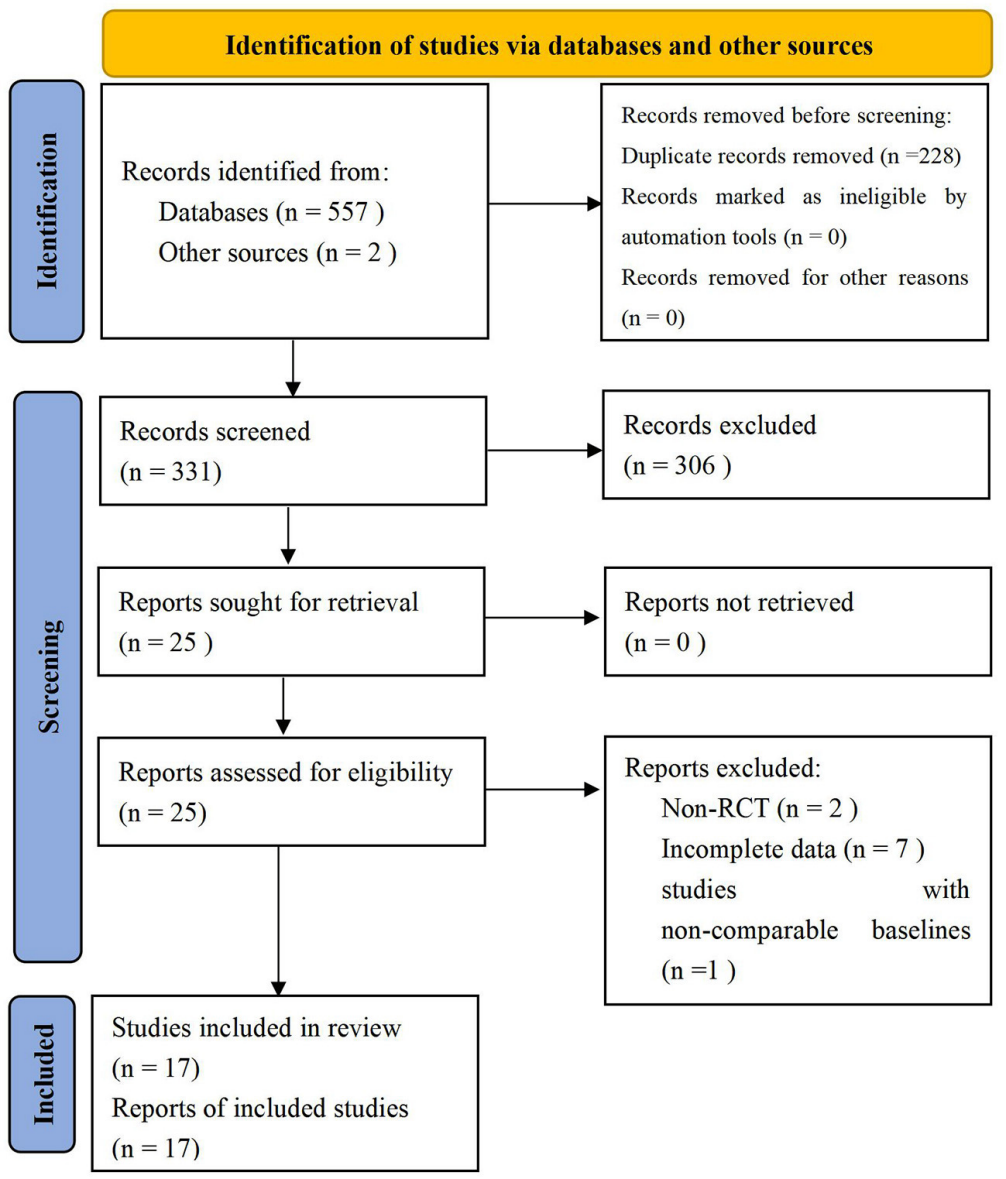
Screening process of the study selection.

A total of 557 studies were obtained by searching the databases, and 2 studies were obtained by reading the review and meta-analysis. First of all, 228 duplicate studies were excluded. Then, 306 studies were excluded by reading the title, abstract, and full text. Finally, a total of 17 studies (Fregni et al., 2006; Kang et al., 2009; Soler et al., 2010; Wrigley et al., 2013; Yilmaz et al., 2014; Ngernyam et al., 2015; Ju et al., 2017; Nardone et al., 2017; Thibaut et al., 2017; Yin and Shi, 2018; Guo et al., 2019; He et al., 2019; Sun et al., 2019; Yang, 2019; Liu et al., 2020; Zhao et al., 2020; Yeh et al., 2021) were included, including 11 in English (Fregni et al., 2006; Kang et al., 2009; Soler et al., 2010; Wrigley et al., 2013; Yilmaz et al., 2014; Ngernyam et al., 2015; Nardone et al., 2017; Thibaut et al., 2017; Sun et al., 2019; Zhao et al., 2020; Yeh et al., 2021) and 6 in Chinese (Ju et al., 2017; Yin and Shi, 2018; Guo et al., 2019; He et al., 2019; Yang, 2019; Liu et al., 2020). Figure 1 shows the screening process of the included studies, and Table 1 shows the characteristics of the included study.

**Table 1:** Characteristics of the included study.

**Study**	**Study design**	**Country**	**Sample (Exp/Ctr)**	**Age (Exp/Ctr)**	**Duration (Exp/Ctr)**	**Injured level (Exp/Ctr)**	**Degree of injury (Exp/Ctr)**	**Intervention scheme**	**Intervention length**	**Outcomes**
Fregni et al. (2006)	Randomized parallel controlled	America	11/6	36.6 ± 12.6/ 34.2 ± 15.8	3.7 ± 1.8/ 3.4 ± 1.5 months	Cervical segments, 5; thoracic segments and lumbar segments, 6; cervical segments, 4; thoracic segments and lumbar segments, 2	Complete injury/incomplete injury, 8/3; complete injury/incomplete injury, 3/3	tDCS, 2 mA, 20 min, the anode electrode is placed over C3 or C4 of the primary motor cortex and the cathode electrode over the contralateral supraorbital area	1 time per day for 5 days	VAS, BDI
Soler et al. (2010)	Randomized parallel controlled	Spain	10/10	40.9 ± 10.8/ 45.0 ± 10.9	8.6 ± 7.3/ 8.6 ± 5.6 years	Cervical segments, 1; thoracic segments and lumbar segments, 9; cervical segments, 4; thoracic segments and lumbar segments, 6	Complete injury/ incomplete injury, 8/2; complete injury/ incomplete injury, 8/2	tDCS, 2 mA, 20 min, the anode electrode is placed over C3 or C4 of the primary motor cortex and the cathode electrode over the contralateral supraorbital area	1 time per day, 5 times per week for 2 weeks	NRS
Wrigley et al. (2013)	Randomized crossed controlled	Australia	10	56.1 ± 14.9	21.3 ± 13.8 years	Thoracic segments, 10	All complete injury	tDCS, 2 mA, 20 min, the anode electrode is placed over C3 or C4 of the primary motor cortex and the cathode electrode over the contralateral supraorbital area	1 time per day for 5 days, the intervention was exchanged between two groups after 4 weeks of elution	NRS, BDI
Ngernyam et al. (2015)	Randomized crossed controlled	Thailand	20	44.5 ± 9.16	54.7 ± 38.7 months	Cervical segments, 7; thoracic segments, 12; lumbar segments, 1	Complete injury/ incomplete injury, 9/11	tDCS, 2 mA, 20 min, the anode electrode is placed over C3 or C4 of the primary motor cortex and the cathode electrode over the contralateral shoulder area	1 treatment, the intervention was exchanged between two groups after 1 week of elution	NRS
Thibaut et al. (2017)	Randomized parallel controlled	America	16/17	51.4 ± 14.9/ 51.0 ± 10.1	5.8 ± 6.3/ 4.6 ± 3.5 years	Not provided	Not provided	tDCS, 2 mA, 20 min, the anode electrode is placed over C3 or C4 of the primary motor cortex and the cathode electrode over the contralateral supraorbital area	1 time per day for 5 days	VAS
Liu et al. (2020)	Randomized parallel controlled	China	12/6	39.9 ± 11.7/ 37.5 ± 14.7	4.7 ± 3.9/ 2.1 ± 1.7 months	Cervical segments, 9; thoracic segments, 3; cervical segments, 4; thoracic segments, 2	Complete injury/incomplete injury, 3/9; complete injury/incomplete injury, 2/4	tDCS, 2 mA, 20 min, the anode electrode is placed over C3 or C4 of the primary motor cortex and the cathode electrode over the contralateral supraorbital area	1 time per day for 5 days	VAS
Yeh et al. (2021)	Randomized parallel controlled	Taiwan, China	6/6	47.3 ± 9.1/ 48.8 ± 14.4	18.5 ± 9.4/ 36.0 ± 39.6 months	Cervical segments, 3; thoracic segments, 2; lumbar segments, 1; cervical segments, 5; thoracic segments, 1	Complete injury/ incomplete injury, 2/4; complete injury/ incomplete injury, 2/4	tDCS, 2 mA, 20 min, the anode electrode is placed over C3 or C4 of the primary motor cortex and the cathode electrode over the contralateral supraorbital area	2 to 3 times per week, 4–6 weeks, 12 times	NRS
Kang et al. (2009)	Randomized crossed controlled	Korea	11	54.8 ± 13.7	60.5 ± 62.4 years	Cervical segments, 5, thoracic segments, 6	Complete injury/ incomplete injury, 3/8	rTMS, 10 Hz, 1000 pulses, 80% resting motion threshold, primary motor cortex	1 time per day for 5 days, the intervention was exchanged between two groups after 12 weeks of elution	NRS
Yilmaz et al. (2014)	Randomized parallel controlled	Turkey	9/7	40.0 ± 5.1/ 36.9 ± 8.0	32.3 ± 25.9/ 35.4 ± 17.9 months	Thoracic segments, 15, lumbar segments, 1	Complete injury/ incomplete injury, 4/5; complete injury/ incomplete injury, 4/3	rTMS, 10 Hz, 1,500 pulses, 110% resting motion threshold, primary motor cortex	1 time per day for 10 days	VAS
Nardone et al. (2017)	Randomized parallel controlled	Austria	6/6	43.0 ± 13.0	9.8 ± 5.0/ 9.0 ± 3.7 years	Cervical segments, 4; thoracic segments, 2; cervical segments, 4; thoracic segments, 2	Complete injury/ incomplete injury, 1/5; complete injury/ incomplete injury, 1/5	rTMS, 10 Hz, 1,250 pulses, 120% resting motion threshold, dorsolateral prefrontal cortex	5 times per week, for 2 weeks	VAS
Ju et al. (2017)	Randomized parallel controlled	China	17/15	39.1 ± 8.5/ 38.5 ± 7.9	3.4 ± 1.9/ 3.5 ± 1.8 months	Thoracic segments, 7; lumbar segments, 10; thoracic segments, 8; lumbar segments, 7	All incomplete injury	rTMS, 10 Hz, 1,400 pulses, 80% resting motion threshold, primary motor cortex	1 time per day, 6 times per week, for 4 weeks	VAS
Yin and Shi (2018)	Randomized parallel controlled	China	30/30	39.6 ± 8.9/ 37.5 ± 8.3	8.1 ± 3.9/ 8.4 ± 4.2 months	Not provided	Complete injury/ incomplete injury, 5/25; complete injury/ incomplete injury, 8/22	rTMS, 20 Hz, 18,000 pulses, 80% resting motion threshold, primary motor cortex	1 time per day, 5 times per week, for 6 months	VAS
He et al. (2019)	Randomized parallel controlled	China	15/15	37.0 ± 11.2/ 35.3 ± 10.3	9.7 ± 3.7/ 9.1 ± 3.7 months	Cervical segments, 6; thoracic segments, 8; lumbar segments, 1; cervical segments, 7; thoracic segments, 7; lumbar segments, 1	All incomplete injury	rTMS, 10 Hz, primary motor cortex	1 time per day, 6 times per week, for 6 weeks	VAS, HAMD
Guo et al. (2019)	Randomized parallel controlled	China	30/30	36.4 ± 12.8/ 36.0 ± 9.7	4.8 ± 1.6/ 4.9 ± 1.3 months	Cervical segments, 21; thoracic segments, 20; lumbar segments, 19	Complete injury/ incomplete injury, 17/43	rTMS, 10 Hz, 80% resting motion threshold, primary motor cortex	5 times per week, for 6 weeks	VAS, HAMD
Yang (2019)	Randomized parallel controlled	China	24/26	35.5 ± 10.0/ 36.2 ± 11.3 ±	15.6 ± 2.5/ 16.8 ± 2.7 months	Cervical segments, 8; thoracic segments, 13; lumbar segments, 3; cervical segments, 13; thoracic segments, 10; lumbar segments, 3	All incomplete injury	rTMS, 10 Hz, 80–120% resting motion threshold, primary motor cortex	5 times per week, for 4 weeks	VAS, HAMD
Sun et al. (2019)	Randomized parallel controlled	China	11/6	45.9 ± 24.6/ 36.0 ± 26.7	Not provided	Cervical segments, 4; thoracic segments, 5; lumbar segments, 2; cervical segments, 1; thoracic segments, 4; lumbar segments, 1	Complete injury/ incomplete injury, 8/3; complete injury/ incomplete injury, 4/2	rTMS, 10 Hz, 1,200 pulses, 80% resting motion threshold, primary motor cortex	1 time per day, 6 times per week for 6 weeks	NRS
Zhao et al. (2020)	Randomized parallel controlled	China	24/24	41.6 ± 9.0	Not provided	Not provided	Complete injury/ incomplete injury, 37/11	rTMS, 10 Hz, 1,500 pulses, 90% resting motion threshold, primary motor cortex	1 time per day, 6 times per week, for 3 weeks	NRS, BDNF, NGF

### Quality and Risk-of-Bias Assessment

**Figure 2:**
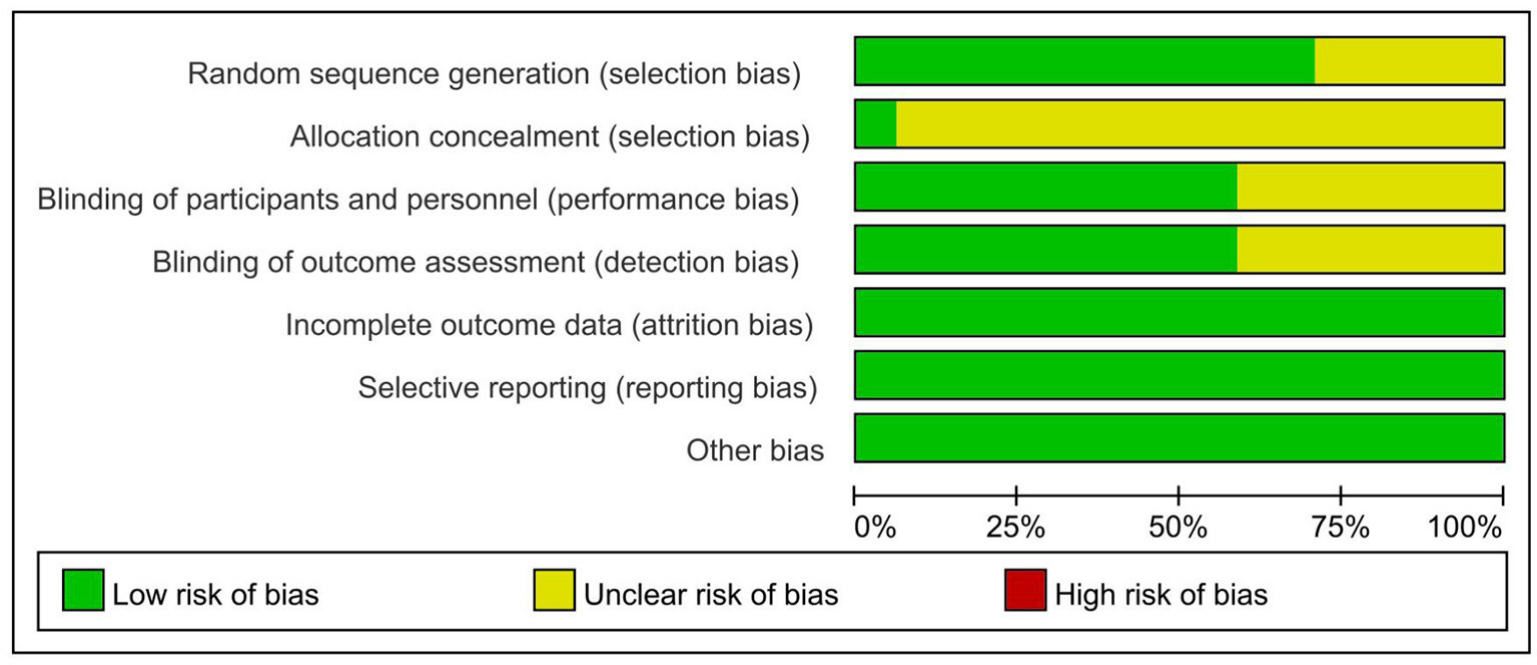
Risk assessment of bias.

All the included studies were random, but 5 studies (Wrigley et al., 2013; Nardone et al., 2017; Thibaut et al., 2017; Yin and Shi, 2018; Zhao et al., 2020) did not mention specific random methods [only 1 study (Yeh et al., 2021) hid the allocation scheme]. A total of 10 studies (Fregni et al., 2006; Kang et al., 2009; Soler et al., 2010; Wrigley et al., 2013; Yilmaz et al., 2014; Ngernyam et al., 2015; Thibaut et al., 2017; Sun et al., 2019; Zhao et al., 2020; Yeh et al., 2021) claimed to be double-blind, and the results of 10 studies (Fregni et al., 2006; Kang et al., 2009; Wrigley et al., 2013; Yilmaz et al., 2014; Nardone et al., 2017; Thibaut et al., 2017; He et al., 2019; Sun et al., 2019; Zhao et al., 2020; Yeh et al., 2021) were measured by blind methods. Details on incomplete outcome data, selective reporting, and other biases are shown in Figure 2 and Table 2. There were 12 high-quality studies (Kang et al., 2009; Wrigley et al., 2013; Yilmaz et al., 2014; Ngernyam et al., 2015; Nardone et al., 2017; He et al., 2019; Sun et al., 2019; Zhao et al., 2020; Yeh et al., 2021) and 5 medium-quality studies (Ju et al., 2017; Yin and Shi, 2018; Guo et al., 2019; Yang, 2019; Liu et al., 2020) with an average score of 7.53.

**Table 2:** Physiotherapy evidence database scores of the included studies.

**Study**	**1**	**2**	**3**	**4**	**5**	**6**	**7**	**8**	**9**	**10**	**11**	**Score**	**Quality grade**
Fregni et al. (2006)	Yes	1	0	1	1	0	1	1	1	1	1	8	High
Soler et al. (2010)	Yes	1	0	1	1	1	0	1	1	1	1	8	High
Wrigley et al. (2013)	Yes	1	0	1	1	0	1	1	1	1	1	8	High
Ngernyam et al. (2015)	Yes	1	0	1	1	1	0	1	1	1	1	8	High
Thibaut et al. (2017)	Yes	1	0	1	1	0	1	1	1	1	1	8	High
Liu et al. (2020)	Yes	1	0	1	0	0	0	1	1	1	1	6	Medium
Yeh et al. (2021)	Yes	1	1	1	1	1	1	1	1	1	1	10	High
Kang et al. (2009)	Yes	1	0	1	1	1	1	1	1	1	1	9	High
Yilmaz et al. (2014)	Yes	1	0	1	1	0	1	1	1	1	1	8	High
Nardone et al. (2017)	Yes	1	0	1	0	0	1	1	1	1	1	7	High
Ju et al. (2017)	Yes	1	0	1	0	0	0	1	1	1	1	6	Medium
Yin and Shi (2018)	Yes	1	0	1	0	0	0	1	1	1	1	6	Medium
He et al. (2019)	Yes	1	0	1	0	0	1	1	1	1	1	7	High
Guo et al. (2019)	Yes	1	0	1	0	0	0	1	1	1	1	6	Medium
Yang (2019)	Yes	1	0	1	0	0	0	1	1	1	1	6	Medium
Sun et al. (2019)	Yes	1	0	1	1	0	1	1	1	1	1	8	High
Zhao et al. (2020)	Yes	1	0	1	1	1	1	1	1	1	1	9	High

### Meta-Analysis

#### Pain Score

**Figure 3:**
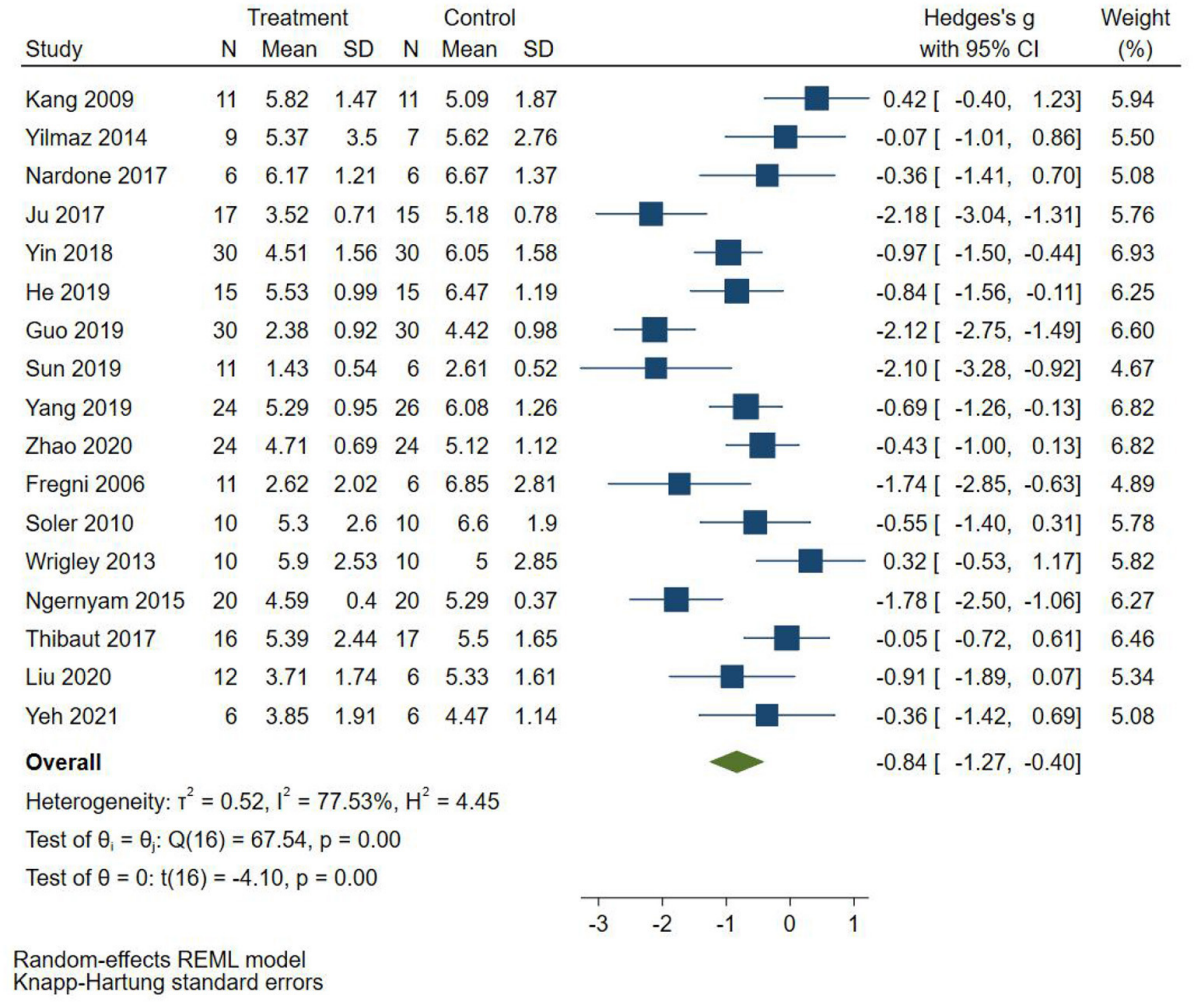
Effect of non-invasive brain stimulation on pain score in patients with neuropathic pain after spinal cord injury.

**Figure 4:**
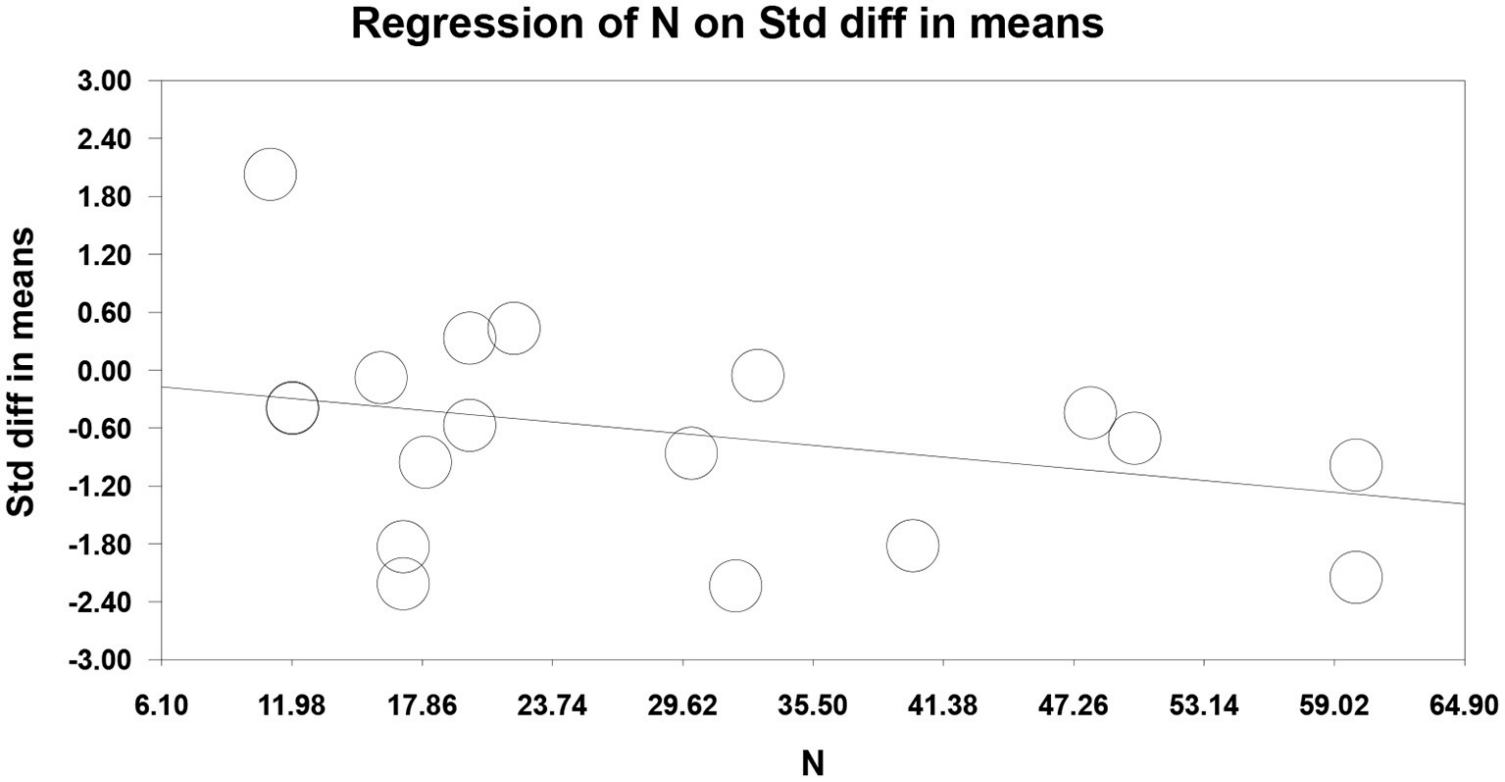
Meta-regression of non-invasive brain stimulation on pain score in patients with neuropathic pain after spinal cord injury.

Seventeen RCTs (Fregni et al., 2006; Kang et al., 2009; Soler et al., 2010; Wrigley et al., 2013; Yilmaz et al., 2014; Ngernyam et al., 2015; Ju et al., 2017; Nardone et al., 2017; Thibaut et al., 2017; Yin and Shi, 2018; Guo et al., 2019; He et al., 2019; Sun et al., 2019; Yang, 2019; Liu et al., 2020; Zhao et al., 2020; Yeh et al., 2021) reported pain score, 10 RCTs (Fregni et al., 2006; Yilmaz et al., 2014; Ju et al., 2017; Nardone et al., 2017; Thibaut et al., 2017; Yin and Shi, 2018; Guo et al., 2019; He et al., 2019; Yang, 2019; Liu et al., 2020) used VAS to evaluate pain, and 7 RCTs (Kang et al., 2009; Soler et al., 2010; Wrigley et al., 2013; Ngernyam et al., 2015; Sun et al., 2019; Zhao et al., 2020; Yeh et al., 2021) used NRS to evaluate pain. SMD was selected as the effect value. The meta-analysis (Figure 3) showed that the pain score of the NIBS group was lower than that of the control group (SMD = −0.84, 95%CI −1.27–−0.40, *P* = 0.00). The meta-regression (Figure 4) showed that heterogeneity was related to sample size (coefficient = −0.02064, *P* = 0.00033). The subgroup analysis (Table 3) showed that the pain scores of the rTMS group were lower than those of the control group (SMD = −0.92, 95%CI −1.56–−0.28, *P* = 0.01), while the tDCS group was not statistically significant compared with those in the control group (SMD = −0.70, 95%CI −1.45–0.04, *P* = 0.06).

**Table 3:** Subgroup analysis of non-invasive brain stimulation for neuropathic pain after spinal cord injury.

**Subgroup analysis**		**Studies**	**SMD (95% CI)**	***P***	**Hartung–Knapp–Sidik–Jonkman (HKSJ) SMD (95% CI)**	**HKSJ *P***
Pain score						
Intervention	tDCS	7	−0.70 (−1.31, −0.10)	0.02	−0.70 (−1.45, 0.04)	0.06
	rTMS	10	−0.92 (−1.47, −0.38)	0.00	−0.92 (−1.56, −0.28)	0.01
Follow-up pain score						
Intervention	tDCS	5	−0.45 (−0.78, −0.12)	0.01	−0.45 (−0.93, 0.02)	0.06
	rTMS	3	−0.18 (−0.50, 0.15)	0.29	−0.18 (−0.46, 0.10)	0.18
Depression score						
Intervention	tDCS	2	−0.05 (−0.67, 0.58)	0.88	−0.05 (−1.02, 0.92)	0.65
	rTMS	4	−0.56 (−0.91, −0.20)	0.00	−0.56 (−1.23, 0.12)	0.08

**Figure 5:**
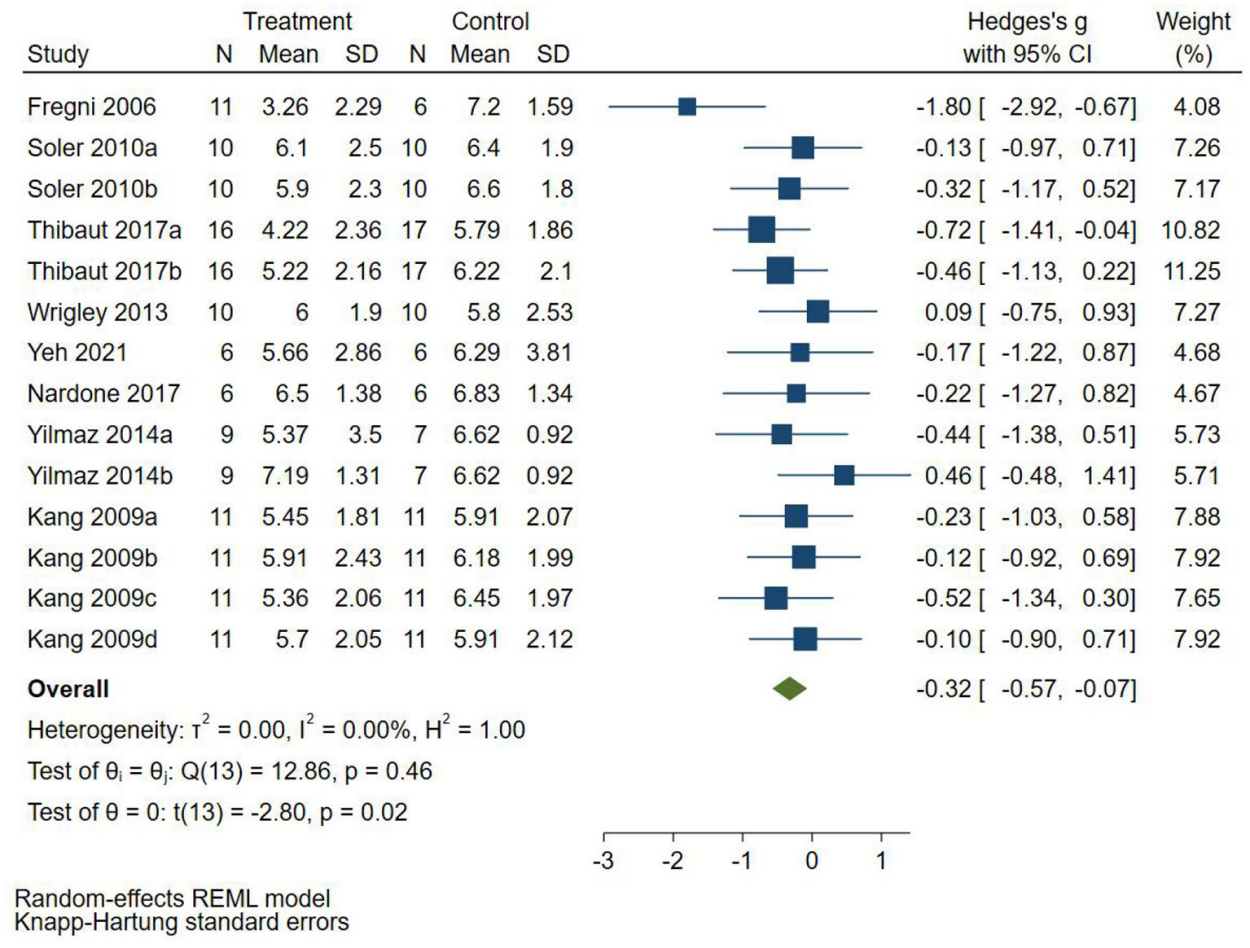
Effect of non-invasive brain stimulation on follow-up pain score in patients with neuropathic pain after spinal cord injury.

Eight RCTs (Fregni et al., 2006; Kang et al., 2009; Soler et al., 2010; Wrigley et al., 2013; Yilmaz et al., 2014; Nardone et al., 2017; Thibaut et al., 2017; Yeh et al., 2021) reported follow-up pain score, 4 RCTs (Fregni et al., 2006; Yilmaz et al., 2014; Nardone et al., 2017; Thibaut et al., 2017) used VAS to evaluate pain, and 4 RCTs (Soler et al., 2010; Wrigley et al., 2013) used NRS to evaluate pain. SMD was selected as the effect value. The meta-analysis (Figure 5) showed that the follow-up pain score of the NIBS group was lower than that of the control group (SMD = −0.32, 95%CI −0.57–−0.07, *P* = 0.02). The subgroup analysis (Table 3) showed that the follow-up pain scores of the tDCS group and the rTMS group were not statistically significant compared with those in the control group (SMD = −0.45, 95%CI −0.93–0.02, *P* = 0.06 and SMD = −0.18, 95%CI −0.46–0.10, *P* = 0.18, respectively).

#### Depression Score

**Figure 6:**
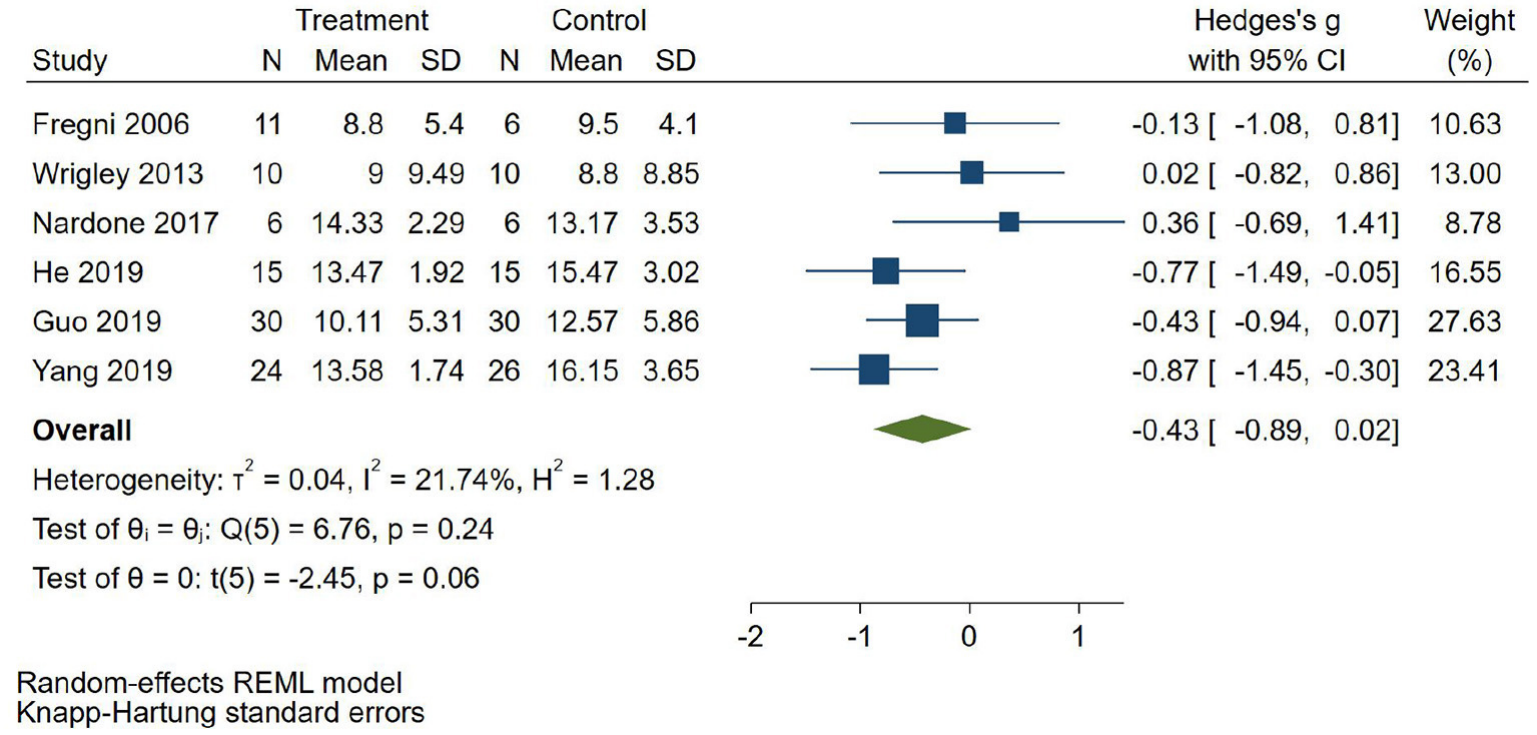
Effect of non-invasive brain stimulation on depression score in patients with neuropathic pain after spinal cord injury.

Six RCTs (Fregni et al., 2006; Wrigley et al., 2013; Guo et al., 2019; He et al., 2019; Yang, 2019) reported depression score, 2 RCTs (Fregni et al., 2006; Wrigley et al., 2013) used BDI to evaluate depression, and 4 RCTs (Nardone et al., 2017; Guo et al., 2019; He et al., 2019; Yang, 2019) used HAMD to evaluate depression. SMD was selected as the effect value. The meta-analysis (Figure 6) showed that the depression score of the NIBS group was not statistically significant than that of the control group (SMD = −0.43, 95%CI −0.89–0.02, *P* = 0.06). The subgroup analysis (Table 3) showed that the follow-up pain scores of the tDCS group and the rTMS group were not statistically significant compared with those in the control group (SMD = −0.05, 95%CI −0.67–0.58, *P* = 0.65 and SMD = −0.56, 95%CI −0.91–0.12, *P* = 0.08, respectively).

#### Other Secondary Outcomes

The study of Zhao et al. (2020) showed that the serum BDNF and NGF increased after rTMS intervention, and the difference was statistically significant compared with the control group.

### Network Meta-Analysis

#### Evidence Network

**Figure 7:**
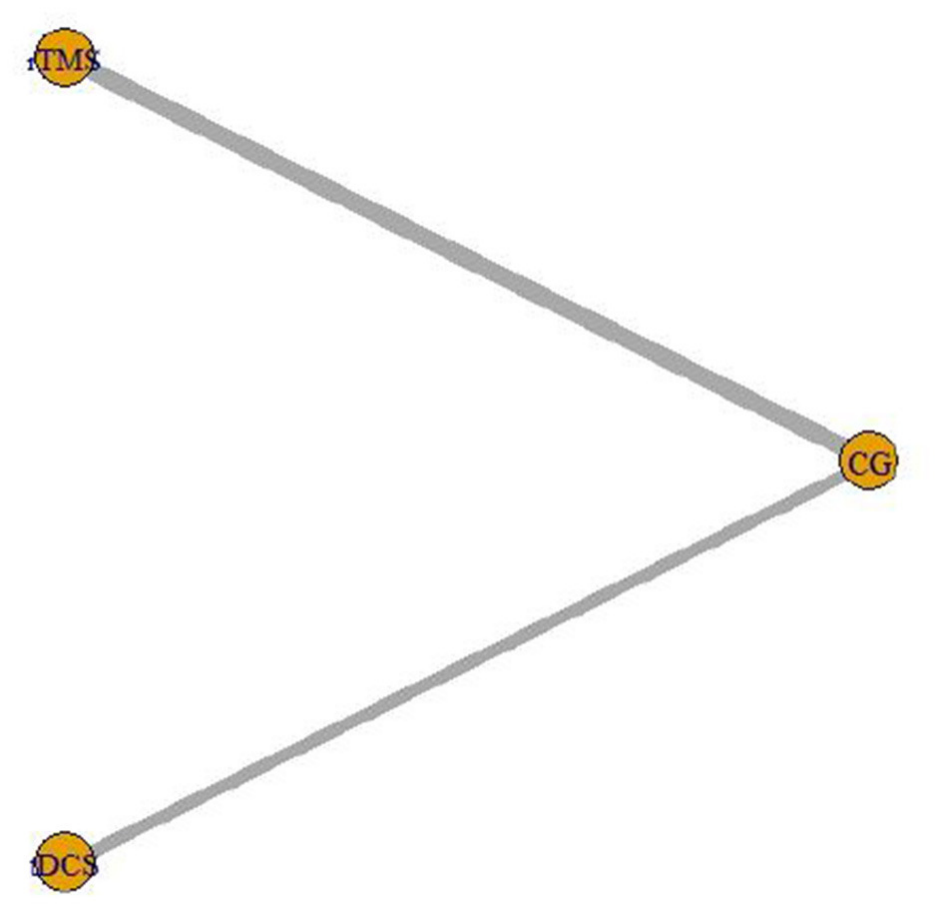
Network relationship of efficacy comparison of different non-invasive brain stimulations.

The intervention of 7 included studies (Fregni et al., 2006; Soler et al., 2010; Wrigley et al., 2013; Ngernyam et al., 2015; Thibaut et al., 2017; Liu et al., 2020; Yeh et al., 2021) was tDCS, and the intervention of 10 (Kang et al., 2009; Yilmaz et al., 2014; Ju et al., 2017; Nardone et al., 2017; Yin and Shi, 2018; Guo et al., 2019; He et al., 2019; Yang, 2019; Zhao et al., 2020) was rTMS. The network relationship of the efficacy comparison of different NIBS is shown in Figure 7. The gray line between each ball represents the RCT, and the two interventions are directly compared. The width of the gray line represents the number of RCTs.

#### Consistency Test

There was no closed loop between the interventions in this study, so there was no need for a consistency test.

#### Convergence Diagnosis

**Figure 8:**
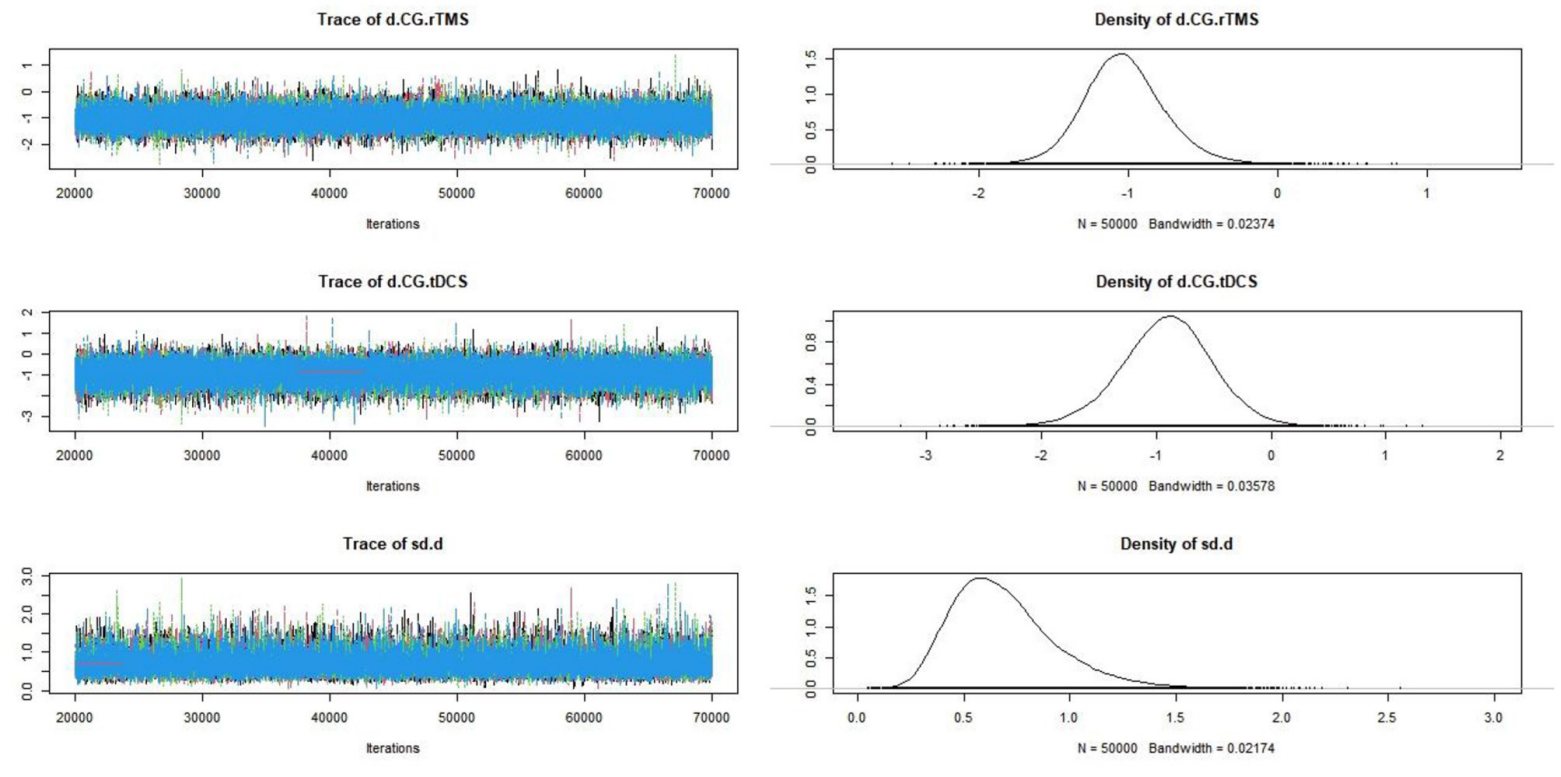
Track density plot of the pain score.

Convergence diagnosis was conducted for the included studies (Figure 8). The bandwidth value was close to 0, indicating good convergence.

#### Probability Ranking

**Figure 9:**
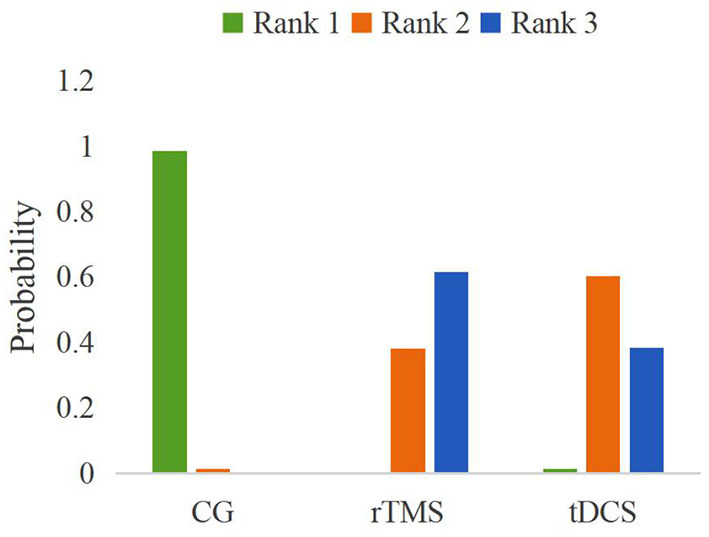
A probabilistic ranking of different non-invasive brain stimulations on pain scores in patients with neuropathic pain after spinal cord injury.

The probability ranking of the network meta-analysis is shown in Figure 9 and Table 4. Rank *N* was the best probability ranking for the negative score of pain score. The greater rank *N* value indicated that the ranking was better. The optimal probability order of the effects of two different NIBS on pain score was rTMS (*P* = 0.62) > tDCS (*P* = 0.38).

**Table 4:** Best probability ranking.

**Intervention**	**Probability**
tDCS	0.38
rTMS	0.62
Control group	0

### Adverse Reactions

Five studies reported that the patients suffered from mild headaches (Fregni et al., 2006; Soler et al., 2010; Wrigley et al., 2013; Guo et al., 2019) and erythema of electrodes (Wrigley et al., 2013; Ngernyam et al., 2015) after NIBS intervention, and the symptoms were relieved after adjusting the stimulation intensity. Adverse reactions were not reported in other studies.

### Publication Bias

**Figure 10:**
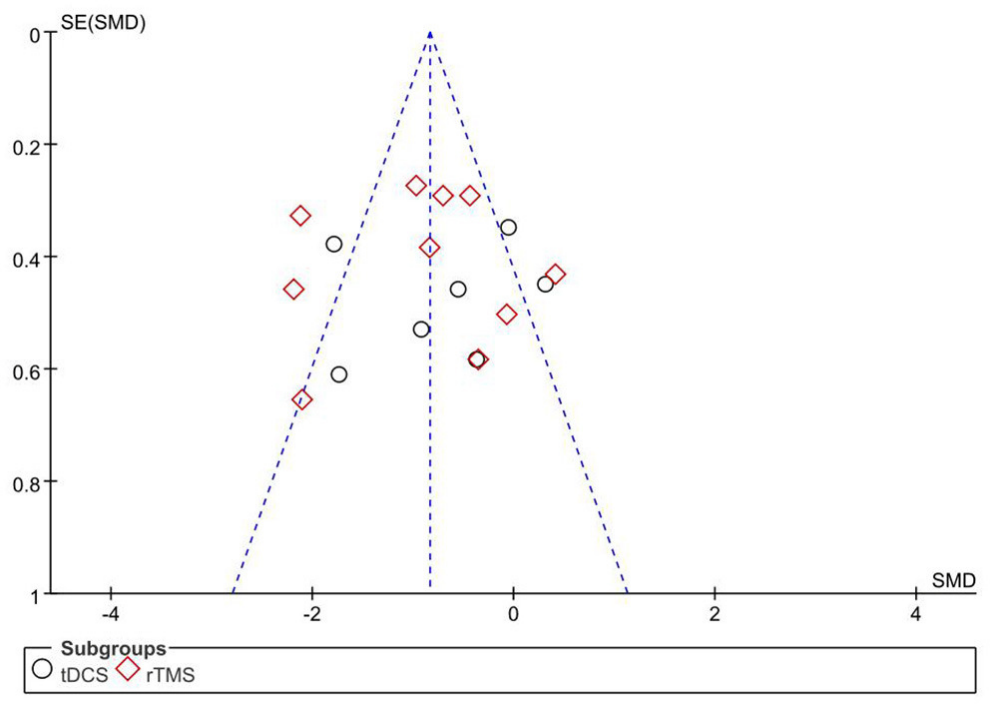
Funnel plot of the included studies.

The primary outcome, pain score, was used as an indicator, and the included studies were analyzed by an inverted funnel plot (Figure 10). GRADE evidence quality evaluation table was shown in the Supplementary Material.

## Discussion

As a new neuromodulation technique, NIBS has been reviewed in relieving NP after SCI (Nardone et al., 2014; Meeker et al., 2020). This article objectively evaluated the rehabilitation of NIBS on NP after SCI from evidence-based medicine and compared the curative effect differences of two different NIBS. In the included studies, VAS or NRS was used to evaluate the pain of the patients, and BDI or HAMD was used to evaluate depression. All scores were negative. The lower score indicated the better result. Our study showed that NIBS could effectively relieve the pain of NP patients after SCI compared with the control group.

Chronic pain is one of the most intractable problems after SCI, including musculoskeletal pain, visceral pain, and NP. Among them, NP is the most difficult to treat (Jetté et al., 2013). According to reports, NIBS is used to control chronic NP, such as post-stroke pain and trigeminal neuralgia, and it has been gradually used in NP after SCI in recent years (O'Connell et al., 2018). In the included studies, the primary motor cortex is the main site of NIBS stimulation. It is speculated that the analgesic mechanism of motor cortex stimulation depends on the activation of the motor cortex, the change of plasticity, and the projection of the motor cortex to the brain parts involved in pain treatment, such as the thalamus, anterior cingulate cortex, and periaqueductal gray of brain stem (Garcia-Larrea and Peyron, 2007). There is also evidence that it may be related to the increased secretion of endogenous opioids (Moisset et al., 2016). Ju et al. (2017) believe that the analgesic mechanism of NP after SCI may be related to the change of cortical excitability. The increase of the motor cortex excitability enhances the inhibition of the cortex on the thalamus, enhances the integration of pain centers into pain, and reduces the abnormal discharge of neurons, thus alleviating pain (Ju et al., 2017).

The stimulation site of Nardone et al. (2017) was the dorsolateral prefrontal cortex. rTMS intervention may activate the anterior cingulate gyrus and pain control loop and release endogenous opioid substances to achieve the analgesic effect. Studies have shown that rTMS in the prefrontal cortex triggers a series of cascade events in the prefrontal cortex and its adjacent marginal areas, feeds back information to important emotion-regulation areas (including cingulate cortex, orbitofrontal cortex, insular lobe, and hippocampus), and may induce dopamine release in the caudate nucleus (George and Wassermann, 1994). It is worth noting that the prefrontal cortex, after rTMS intervention, also plays an important regulatory role in the frontal cingulate gyrus, which participates in emotional control (Paus et al., 2001). Our study showed that the symptoms of depression tended to be alleviated after rTMS intervention, which was consistent with the results of Nardone et al. (2017). The study of Defrin et al. (2007) showed that the depressive symptoms of the rTMS group and the control group were alleviated, but only the pain threshold in the rTMS group was significantly increased. However, after tDCS intervention, there was no significant difference in depressive symptoms between the two groups, which may be related to a few RCTs included, which needed further study in the future. Depression is generally believed to be closely related to pain. Our study cannot determine the causal relationship between depression and pain improvement, which may be the starting point for future research.

The results of the pain follow-up score showed that these were not statistically significant in the tDCS group and the rTMS group compared with those in the control group. Previous studies (Nardone et al., 2014) have shown that rTMS can temporarily relieve NP. However, there is still a conservative view about its long-term analgesic effect. A meta-analysis (O'Connell et al., 2018) showed that the short-term analgesic effect was significant, but the long-term analgesic effect was poor after rTMS intervention, which was consistent with our study.

In addition, an included study showed that 10-Hz rTMS treatment in the primary motor cortex could reduce the pain intensity of acute NP, accompanied by an increase of BDNF and nerve growth factor secretion (Zhao et al., 2020). More and more studies have shown that the level of BDNF is directly related to the analgesic effect of rTMS. Wang et al. (2011) found that the plasma BDNF levels in rats increased threefold after 5-Hz rTMS intervention. Zhao et al. (2020) believed that rTMS was related to the increase of NGF level, and its main role was to protect the neurons and recover the nerve function.

The results of the network meta-analysis showed that rTMS was superior to tDCS in improving NP after SCI. The two NIBS have similar effects on pain by changing the cortical excitability, but their mechanisms are different. The tDCS causes hyperpolarization or depolarization in the stimulation area, resulting in a weak sustained current, while rTMS induces changes in synaptic enhancement efficiency by long-term enhancement and inhibition mechanisms, resulting in pulses with an intensity close to the threshold (Fregni et al., 2006). However, tDCS can only induce local currents in neurons but cannot lead to spontaneous neuron discharges (Li et al., 2021).

Stimulation frequency and treatment times are important factors that affect the analgesic effect of rTMS. Similarly, the analgesic effect of tDCS is also influenced by the current intensity and electrode size. In the included studies, the resting motion thresholds for rTMS were mainly 80–120% and 5–20 Hz, and the currents for tDCS were mainly 2 mA and 20 min. At present, there are different opinions on specific treatment parameters, such as treatment frequency, current, and stimulation site, and how to prolong the duration of analgesic effect. In this study, the relationship between the intervention length and cumulative intervention time and the relief of NP after SCI has not been determined. There are two types of NP in patients after SCI: one with distribution at the pathological level and the other with more diffuse distribution below the pathological level [33]. Although these two types of NP are severe and persistent, their potential therapeutic mechanisms may be different. Large-scale and multicenter trials are needed in the future to comprehensively evaluate the effect of NIBS on NP after SCI and to explore the mechanism of NIBS by combining functional magnetic resonance imaging and functional near-infrared imaging.

Although this study follows the criteria of the systematic review and network meta-analysis report (PRISMA statement), there are also some limitations. The amount of included studies was small. Our study cannot determine the causal relationship between depression and pain improvement as well as the length of intervention, cumulative intervention time, and NP improvement after SCI. Some included studies do not describe specific random methods, allocation concealment, and blind methods, which may reduce the reliability of the results. The baseline level, intervention scheme, and severity of SCI may affect the meta-analysis.

## Conclusion

Our research shows that NIBS can relieve NP after SCI. The effect of rTMS on NP after SCI is superior to that of tDCS. We suggest that the rTMS parameters are 80–120% resting motion threshold and 5–20 Hz while the tDCS parameters are 2 mA and 20 min. However, it is necessary to carry out a large-scale, multicenter, double-blind, high-quality RCT to explore the efficacy and mechanism of NIBS for NP after SCI. Besides these, NIBS has no obvious adverse reactions during NP period after SCI, which is worthy of clinical application.

## Data Availability Statement

The original contributions presented in the study are included in the article/Supplementary Material, further inquiries can be directed to the corresponding author.

## Author Contributions

LL designed and wrote this study. HH provided guidance regarding the methodology. YY and TZ reviewed the full manuscript. ZL and XS took part in the data selection and extraction. YJ and FW performed the statistical analysis and analyzed the data. All authors contributed to the article and approved the submitted version.

## Funding

This study was received support from the Shandong Traditional Chinese Medicine Science and Technology Development Planning (No. 2017-018), the Shandong University of Traditional Chinese Medicine Research and Innovation Outstanding Team (No. 220316), and the Shandong Provincial Universities Scientific Research Development Planning (No. J18KB130).

## Conflict of Interest

The authors declare that the research was conducted in the absence of any commercial or financial relationships that could be construed as a potential conflict of interest.

## Publisher's Note

All claims expressed in this article are solely those of the authors and do not necessarily represent those of their affiliated organizations, or those of the publisher, the editors and the reviewers. Any product that may be evaluated in this article, or claim that may be made by its manufacturer, is not guaranteed or endorsed by the publisher.
