# Supplementary material for: Non-invasive Brain Stimulation for Neuropathic Pain After Spinal Cord Injury: A Systematic Review and Network Meta-Analysis
Source: Front Neurosci. 2022 Feb 11;15:800560. doi: 10.3389/fnins.2021.800560 (PMC8873374; doi:10.3389/fnins.2021.800560)
Supplement: Supplementary file 1 [file Table_1.docx]

**Supplement : GRADE evidence quality evaluation table.**

| Outcomes | limitations | inconsistency | indirectness | inaccuracy | publication bias | N | Quality grade |
| --- | --- | --- | --- | --- | --- | --- | --- |
| Pain score | serious | serious | non-significant | non-significant | non-significant | 268/250 | low |
| Depression score | serious | non-significant | non-significant | non-significant | non-significant | 102/98 | medium |
| PSQI | serious | non-significant | non-significant | non-significant | non-significant | 39/41 | medium |
